# Sudden Death from Fright

**Published:** 1883-11

**Authors:** J. E. Cooney


### Selections.

### Sudden Death From Fright.

The following case appears worthy of being placed on record:

G. E. H——, aged seven years, the son of a sweep, had been witnessing a fight between two gipsy lads, when, as the contest became exciting, he suddenly fell backwards and was dead in a few moments. I was called in by the police immediately after death, and found the body, which was still warm, lying on its dorsal aspect on a soft patch of grass. The features had a placid expression, and his soot-smeared face exhibited the traces of recent tears. It was stated that there was no one nearer to the boy when he fell than the two belligerents, who were about ten yards' distance.

The history which I elicited was as follows: The father had been a soldier in India and elsewhere for twenty-one years prior to taking up his present occupation, which he had followed for the last three months only. The mother had died suddenly a year ago, and was the subject of a coroner's inquest; but the cause of her death I have, in vain, tried to discover. The deceased was an only son. His sister, aged four years, seems delicate, and I learn that his half-brother and sister are robust and healthy, and have no neurotic tendency. He was of a remarkably affectionate disposition, and was easily excited by the slightest unusual occurrences. The boy had had his dinner a quarter of an hour previous to death, and had kissed his father before leaving home as usual.

Agreeably to the coroner's order, I made an autopsy forty-eight hours after death. The body was plump, and the external aspect, on examination, showed no marks of injuries or contusions. Head: The dura mater was not adherent to the brain, neither was there any sign of fractures or effusions of fluid. The brain weighed forty-two ounces, was well formed, firm, and with convolutions and sulci well marked. The puncta vasculosa were a little more than ordinarily prominent. The several ganglia and ventricles of the brain were normal; minute examination betrayed no rupture of blood vessels or extravasations. The examination of spine showed no dislocation or other injury in the region of the atlas and dentata or elsewhere. The medulla oblongata and cord were apparently healthy. Chest: The right cavity showed a few old pleuritic adhesions; left pleura and cavity healthy. The lungs were normal, though a little gorged with blood; the pulmonary veins contained some fluid blood; the pericardium was healthy, and its secretion was of the normal color, quality and quantity; the right auricle was much distended, almost to bursting, with dark grumous fluid blood; the right ventricle was about one-third full of the same; the superior and inferior cava and hepatic veins were also much distended with dark fluid blood; the left auricle was full, but not so distended as the right; the left ventricle also contained a little fluid blood. The pulmonary veins were filled with fluid blood, and the two left veins terminated by a common opening in the left auricle. The heart's septum was normal, and its valves competent, and there was no evidence of rupture or disease. The liver was normal and healthy, the hepatic veins being filled. The kidneys were normal. The urinary bladder was half filled, and was perfectly healthy. There was no sign of injury to the abdominal walls. The diaphragm, peritoneum, spleen, pancreas, and supra-renal bodies were perfectly healthy and in their normal positions.

I gave my opinion at the inquest that death in this instance was due to strong emotion, the emotional impulse being transmitted from the brain to the cardio-inhibitory centre in the medulla, and thence along the inhibitory fibres of the vagus to the intra-cardiac ganglia, thus arresting the heart's action (in diastole). I proceeded to make the post mortem examination with a prejudiced mind that perhaps the death was due either to external violence on the head or pit of the stomach, or rupture of the heart or some blood vessel; but after a minute and patient examination, I found that I could only arrive at the opinion already given.—*J. E. Cooney, L. R. C. P., in The Lancet.*

